# Validation of Treatment Discontinuation in Claims Data Using NLP and Electronic Health Records

**DOI:** 10.1093/geroni/igaf122.356

**Published:** 2025-12-31

**Authors:** Chun-Ting Yang, Kerry Ngan, Dae Hyun Kim, Jie Yang, Jun Liu, Joshua Lin

**Affiliations:** Brigham and Women’s Hospital, Boston, Massachusetts, United States; Brigham and Women’s Hospital, Boston, Massachusetts, United States; Hebrew SeniorLife, Boston, Massachusetts, United States; Brigham and Women’s Hospital, Boston, Massachusetts, United States; Brigham and Women’s Hospital, Boston, Massachusetts, United States; Brigham and Women’s Hospital, Boston, Massachusetts, United States

## Abstract

Measuring medication discontinuation in claims data primarily relies on the gaps between prescription fills, but such definitions are rarely validated. This study aimed to establish a natural language processing (NLP)-based framework to validate claims-based discontinuation algorithms for commonly used medications against NLP-based reference standards from electronic health records (EHRs). We identified 35,010 patients receiving antipsychotic medications (APMs), benzodiazepines, warfarin, or direct oral anticoagulants (DOACs) from EHRs at the Mass General Brigham in 2007-2020. These EHR data were linked with 82,455 Medicare Part D claims. An NLP-aided chart review was applied to determine medication discontinuation from EHRs (reference standard). In claims data, we defined discontinuation based on a prescription gap larger than 15-90 days (claims-based algorithms). Sensitivity, specificity, and predictive values of claims-based algorithms against the reference standard were calculated. We found that the sensitivity and specificity of 90-day-gap-based algorithms were 0.49 and 0.82 for haloperidol, 0.47 and 0.86 for atypical APMs, 0.54 and 0.75 for benzodiazepines, 0.40 and 0.81 for warfarin, and 0.46 and 0.89 for DOACs, respectively. The corresponding estimates for 15-day-gap-based algorithms were 0.71 and 0.58 for haloperidol, 0.61 and 0.64 for atypical APMs, 0.75 and 0.45 for benzodiazepines, 0.64 and 0.50 for warfarin, and 0.61 and 0.66 for DOACs, respectively. Positive predictive values were primarily affected by medication discontinuation rates and less by gap lengths. In summary, the accuracy of claims-based discontinuation algorithms differs by medications and prescription gaps. This study demonstrates the scalability and utility of the NLP-based validation framework for multiple medications.

